# Specific Learning Disorder: Prevalence and Gender Differences

**DOI:** 10.1371/journal.pone.0103537

**Published:** 2014-07-29

**Authors:** Kristina Moll, Sarah Kunze, Nina Neuhoff, Jennifer Bruder, Gerd Schulte-Körne

**Affiliations:** Department of Child and Adolescent Psychiatry, Psychosomatics, and Psychotherapy, Ludwig-Maximilians University, Munich, Germany; Utrecht University, Netherlands

## Abstract

Comprehensive models of learning disorders have to consider both isolated learning disorders that affect one learning domain only, as well as comorbidity between learning disorders. However, empirical evidence on comorbidity rates including all three learning disorders as defined by DSM-5 (deficits in reading, writing, and mathematics) is scarce. The current study assessed prevalence rates and gender ratios for isolated as well as comorbid learning disorders in a representative sample of 1633 German speaking children in 3^rd^ and 4^th^ Grade. Prevalence rates were analysed for isolated as well as combined learning disorders and for different deficit criteria, including a criterion for normal performance. Comorbid learning disorders occurred as frequently as isolated learning disorders, even when stricter cutoff criteria were applied. The relative proportion of isolated and combined disorders did not change when including a criterion for normal performance. Reading and spelling deficits differed with respect to their association with arithmetic problems: Deficits in arithmetic co-occurred more often with deficits in spelling than with deficits in reading. In addition, comorbidity rates for arithmetic and reading decreased when applying stricter deficit criteria, but stayed high for arithmetic and spelling irrespective of the chosen deficit criterion. These findings suggest that the processes underlying the relationship between arithmetic and reading might differ from those underlying the relationship between arithmetic and spelling. With respect to gender ratios, more boys than girls showed spelling deficits, while more girls were impaired in arithmetic. No gender differences were observed for isolated reading problems and for the combination of all three learning disorders. Implications of these findings for assessment and intervention of learning disorders are discussed.

## Introduction

Learning disorders are among the most frequently diagnosed developmental disorders in childhood. Epidemiological studies report comparable prevalence rates of 4–9% for deficits in reading and 3–7% for deficits in mathematics (DSM-5; [1]). More recently, studies have started examining the relationship between deficits in different learning domains (i.e., deficits in reading and deficits in mathematics) in order to better understand their overlap, rather than focusing on a single deficit only. Findings suggest that children experiencing a deficit in one learning domain frequently show deficits in other domains as well [2]–[6]. Furthermore, behaviour-genetic analyses provide evidence that reading and mathematics disorders share genetic variance (e.g., [7], [8]). In line with these findings the latest edition of the American Psychiatric Association’s Diagnostic and Statistical Manual of Mental Disorders (DSM-5; [1]) broadened the diagnostic category by using the generic term “Specific Learning Disorder” as overall diagnosis, incorporating difficulties in learning academic skills, such as reading, writing, and mathematics, which have been classified as separate disorders in previous editions (DSM-IV: 315.0; 315.2; 315.1; [9]).

Although comorbidity rates between learning disorders are supposed to be high, it is important to note that deficits in specific learning domains also occur in isolation. Dissociations between learning disorders are not only observed between deficits in literacy and mathematics but also between different literacy components, such as difficulties in decoding (dyslexia) and in reading comprehension (e.g., [10], [11]) and between deficits in reading and in spelling (e.g., [12], [13]). Importantly, these specific deficits are characterized by distinct underlying cognitive causes and therefore require different interventions [14] for review). Thus, it seems crucial to assess the exact nature of the learning disorder in order to ensure adequate treatments. In DSM-5 this issue is taken into account by adding specifiers to the generic diagnosis “Specific Learning Disorder” to provide additional information about the domains that are affected. Three types of Learning Disorders can be coded: deficits in reading, deficits in writing and deficits in mathematics, which can be further specified by detailed descriptions (e.g., reading: deficits in accuracy, fluency, comprehension) and by severity ratings. Compared to most previous studies that focused on the relationship between mathematics and reading, the current study includes all three learning domains and assesses prevalence rates for isolated as well as combined deficits in reading, writing, and mathematics.

The inclusion of severity ratings in DSM-5 reflects the idea that developmental disorders are best conceptualized as dimensional disorders rather than diagnostic categories and should be seen as the outcome of multiple interacting risk factors [14]–[18]. It follows from this idea that any cutoff criteria used to classify learning disorders are somehow arbitrary. Obviously prevalence rates for specific learning disorders reflect the chosen deficit criteria; however, it is not clear how comorbidity rates (i.e., the percentage of all children with a specific learning disorder who also experience deficits in another learning domain) change with varying cutoff criteria, as the empirical basis for comorbidity rates comparing different cutoff-criteria is scarce [2], [4]. Therefore, the current study aimed to examine systematically how comorbidity rates change depending on the chosen cutoff criterion.

Another relevant adaptation in DSM-5, which was taken into account in the current study, is that a predefined discrepancy between IQ and the affected learning domain is no longer required for diagnosing learning disorders. Performance of the affected academic skill has to be well below average for age and not attributable to intellectual disability (defined by IQ below 70). This adaptation is based on research showing that children who fulfill and those who do not fulfill the IQ-discrepancy criterion do not differ in terms of symptomatology, underlying cognitive deficits, and response to intervention (e.g., [19]–[21]).

In sum, comprehensive models of learning disorders have to consider both disorders in specific learning domains as well as comorbidity between learning disorders [17], [22]. Before examining associations and dissociations of learning disorders on a cognitive or neurobiological level, the first question arising is how often isolated and combined learning deficits in reading, writing, and mathematics can be observed on a *behavioural* level. The existing prevalence studies reporting comorbidity rates for learning disorders based on population based samples are summarized in **Table 1**. As evident, surprisingly few studies included all three learning domains [2], [4], [19], [23], and only two of them analysed comorbidity rates based on different cutoff criteria [2], [4]. While studies consistently report higher comorbidity rates than expected given the population based prevalence rates, this overview also shows that comorbidity rates vary widely across studies. The high variability in comorbidity rates might reflect the different tests and criteria used for classification. One methodological problem is that comorbidity rates can be artificially increased due to symptom overlap in the measures used for classification. For example, arithmetic tests which include word problems do not only measure calculation skills but also require reading and comprehension skills. As a consequence, children with reading disorder can be impaired in such tasks, although their calculation skills might be within the normal range. Ideally, measures should be domain specific in order to avoid that additional skills tapping into other learning domains are required. Therefore it is somehow unfortunate that the majority of studies analyzing comorbidity rates used mathematic achievement tests assessing a wide range of mathematic skills, including word problems.

**Table 1:** Overview prevalence studies reporting comorbidity rates for learning disorders based on representative samples.

Study	N	Cutoff	AD	RD	SD	AD+RD	AD+SD	AD of RD	RD of AD	AD of SD	SD of AD
**Badian, 1983**	1476	-	3.6	2.2	-	2.7	-	55	42	-	-
**Lewis et al., 1994**	1056	standard score 85	1.3	3.9	-	2.3	-	37	64	-	-
**Gross-Tsur et al., 1996**	3029	2 step procedure:	5.4	-	-	1.1	-	-	17	-	-
		20^th^ percentile+2 years delay									
**Ostad, 1998**	927	school support services for 2 years	-	-	-	-	5.6	-	-	51	-
**Badian, 1999**	1075	20^th^ percentile	3.9	6.0	-	3.0	-	-	-	-	-
**Barbaresi et al., 2005**	5718	3 formula: (1) regression based,	-	-	-	-	-	-	43–65	-	-
		(2) IQ-discrepancy, (3) low									
		achievement (<90+IQ>80)									
**von Aster et al., 2007**	337	1.5 sd	1.8	3.3	5.7	4.2	4.2	56	70	70	42
**Dirks et al., 2008**	799	25^th^ percentile	10.3	19.9	-	7.6	-	28	43	-	-
		25^th^ percentile	9.0	-	14.5	-	8.1	-	-	47	36
		10^th^ percentile	5.6	8.0	-	1.0	-	11	15	-	-
**Landerl & Moll, 2010**	2586	1 sd	-	-	-	-	-	37	39	37	40
		1.5 sd	3.2	2.9	4.1	1.6	2.3	26	23	26	37
**Fischbach et al., 2013**	2195					**AD+RD+SD**					
(reading comprehension)		T-score 40+IQ≥85	5.0	4.6	5.7	4.2	-	-	-	-	-
		T-score 40+IQ≥1.2 sd	2.6	2.6	4.0	2.0	-	-	-	-	-
**Dhanda et al., 2013**	1156	Not described:	**% of learning disorder**			**comorbid**					
		Learning disorder 12.8%	16	22	22	40	-	-	-	-	-

*Note:* sd = standard deviation; RD = Reading Deficit; SD = Spelling Deficit; AD = Arithmetic Deficit.

Another methodological problem is that cutoff criteria for normal performance are not considered in any of the existing studies. Excluding children scoring in-between the deficit and the normal range would ensure that children categorized as having an isolated deficit did not just miss the cutoff criterion for a second deficit but are indeed unimpaired and score within the normal range in other learning domains.

The second question arising when analyzing learning disorders in a representative sample is how gender ratios differ depending on the affected learning domain. For literacy problems, findings generally suggest higher prevalence rates for boys than girls (see [24] for review); however in English, the language where the majority of studies is carried out, reading and spelling deficits are generally not analysed separately, so that it remains unresolved if gender ratios differ for different literacy components (i.e., reading and spelling deficits). Indeed, when Landerl and Moll [4] analyzed gender ratios in a population based German-speaking sample they reported balanced gender ratios for reading (fluency) deficits, but a disproportionate number of boys for spelling deficits. With respect to mathematics disorder findings are mixed, with the majority of studies reporting balanced gender ratios [2], [5], [6] or a higher rate among females [4], [19], [25]; however, a few studies found the opposite pattern and reported a higher rate of mathematics disorder for boys than for girls [26], [27].

The current study aimed to assess prevalence rates for isolated as well as comorbid learning disorders in a representative sample of 1633 German speaking children in 3^rd^ and 4^th^ Grade. The study was designed to overcome some of the shortcomings reported above: First, all three learning domains, as stated in DSM-5, were included: reading (reading fluency), writing (spelling), and mathematics (calculation). In addition, comorbidity rates were analysed for three different deficit criteria (1, 1.25 and 1.5 standard deviations below the age-specific mean), and by including cutoff-criteria for normal performance in order to clearly differentiate between isolated and comorbid deficits. Artificially induced comorbidity was avoided by (1) analyzing comorbidity rates in a representative rather than a clinical sample where comorbidity rates might be inflated (sampling bias), (2) by applying standardized tests administered by objective testers rather than using teacher or parental ratings (rater bias), and (3) by using domain specific tests that do not require additional skills associated with other learning domains (symptom overlap). Finally, the study aimed to assess gender ratios in isolated as well as comorbid deficit groups.

## Method

### Participants

Participants were recruited from 119 classrooms in 18 primary schools from 8 districts in and around Munich. Information letters were sent out through schools to all parents of children in the participating schools. Out of all children invited, 61% participated in the study. The schools were chosen out of 132 existing schools and were distributed all over Munich in order to include a mix of small rural and large urban schools and to take account for the differences in socioeconomic status (SES) between the 25 existing districts in Munich. Information about SES for each district was obtained from the social service department of Munich. The SES score consists of five scales (unemployment benefit, housing benefit, percentage of migrants, child support benefit, child protection), which are scored on a scale between 1 and 3, with higher scores indicating more needs for support and therefore reflecting lower SES. The mean SES score for the participating school districts was 1.6 and did not differ significantly (*t* = 0.68, *p*>.05) from the mean score for the whole region of Munich (1.7), indicating that the SES of the current sample is representative for the region. In addition, the percentage of migrants in the 8 districts was compared to the mean percentage of migrants for all 25 districts in Munich. No significant difference was found (Mean: 21% vs. 23%; *t* = 1.63; *p*>.05). The majority of migrants in Munich (75%) are from Europe and Turkey, which is highly similar to the statistics reported by the government for Germany (about 79%).

In total 1633 children from 3^rd^ (896) and 4^th^ (737) grades (50.6% male) took part in the study. Ethical approval was granted by the ethics committee of the Department of Child and Adolescent Psychiatry, Psychosomatic, and Psychotherapy, Philipps University Marburg; informed written consent was given by caregivers. Attendance rates in classrooms ranged between 19% and 100% with a mean attendance rate of 60% (standard deviation = 19). To ensure that prevalence rates are not systematically influenced by classrooms with relatively low attendance rates, prevalence rates for deficits in reading, spelling and arithmetic (for all three deficit criteria) were reanalysed in a subsample excluding 19 classes with low attendance rates (<41%, which corresponds to one standard deviation below the mean attendance rate). In this subsample the percentages of affected children were not significantly different from those observed for the whole sample (all *t*s<1, *p*s>.05), speaking against a systematic sampling bias caused by differences in attendance rates.

### Measures and Procedures

Reading, spelling and arithmetic skills were measured using standardized classroom tests. All tests were administered and scored by trained research assistants according to the manual. Following DSM-5, we did not apply an IQ-discrepancy criterion when classifying learning disorders. Although IQ was not measured in the current sample, it has to be noted that in the German school system children with intellectual disabilities generally visit special schools, which were not included in the current study. Therefore, it can be assumed that in line with the DSM-5 criteria, deficits identified in the different learning domains in this sample are specific rather than the consequence of general intellectual disabilities.

A restriction of the current procedure is that clinical criteria for classifying learning disorders could not be applied; first, children were assessed in classrooms rather than tested individually and secondly, children were assessed at one time point only, so that information about the persistence of learning problems is not available. Thus, the term learning disorder in the current context refers to low performance in one or several learning domains rather than to a clinical diagnosis of learning disorder.

#### Reading

A standardized sentence reading test measuring reading fluency was administered (SLS; [28]). In more consistent orthographies like German, reading fluency is the appropriate measure to assess individual differences in word recognition as reading accuracy is close to ceiling after one year of reading instruction. Children had to silently read single sentences as quickly as possible and indicate if their meaning is correct by encircling a checkmark or cross printed next to each sentence. In order to measure reading fluency rather than comprehension, semantic content of the sentences is kept simple (e.g., dogs can bark), resulting in generally negligible rates of incorrect responses or omissions (4.5% in the standardization sample). The test score is the number of correctly marked sentences within three minutes.

#### Spelling

A standardized spelling test for 3^rd^ and 4^th^ graders (DRT 3 and DRT 4; [29], [30]) was administered. Children had to fill-in 44 (DRT 3: diagnostic spelling test for 3^rd^ graders) or 42 (DRT 4: diagnostic spelling test for 4^th^ graders) single words which were dictated in a sentence frame. The percentage of correctly spelled words was calculated.

#### Arithmetic

Basic calculation skills (addition, subtraction, multiplication, and division) were assessed using four subtests of a standardized arithmetic test (HRT; [31]). In each subtest children were instructed to solve as many calculations as possible (max. 40 per subtest) within two minutes and write down the correct answers. All subtests start with simple calculations including only single digits as operands (e.g., 1+6 = _; 5–3 = _; 2×2 = _; 6∶2 = _), and increase in difficulty (e.g., 77+45 = _; 120–22 = _; 11×14 = _; 124∶4 = _), so that the same test can be used for grades 1 to 4. For each subtest, an efficiency score was calculated based on the number of correctly solved items within the two-minute time limit. For the analyses a composite efficiency score for the four subtests based on z-scores was calculated.

### Data analyses

In order to combine 3^rd^ and 4^th^ Grade data for the analyses, grade-specific z-scores were calculated for each measure. Based on the whole sample (N = 1633), prevalence rates and gender ratios were analysed for isolated disorders, that is Reading Disorder (RD), Spelling Disorder (SD), and Arithmetic Disorder (AD) and for combined learning disorders, namely Reading and Spelling Disorder (RD+SD), Reading and Arithmetic Disorder (RD+AD), Spelling and Arithmetic Disorder (SD+AD) and Reading, Spelling, and Arithmetic Disorder (RD+SD+AD). Analyses were calculated for three different deficit criteria (1, 1.25, and 1.5 standard deviations below the grade-specific mean).

One problem of this approach is that children who just missed the cutoff criterion for a second deficit (e.g. scored 0.99 standard deviations below the mean when using a deficit criterion of 1 standard deviation) are categorized as having an isolated learning disorder, although their performance in the second domain is similarly poor. In order to ensure that children with an isolated deficit are indeed unimpaired in the other learning domains, we reran the analyses including an additional cutoff criterion for normal performance, so that children who just missed the deficit criterion and scored above the deficit criterion but below the criterion for normal performance were excluded. Normal performance was defined by a score within or above the normal range which corresponds to a score of ≥−1 standard deviation. When applying a deficit criterion of 1 standard deviation the cutoff for the normal range had to be increased (≥−0.85 standard deviations) in order to allow a clear distinction between impaired and normal performance. As a consequence, normal performance was defined by a score of ≥−0.85 standard deviations when applying the 1 standard deviation deficit criterion and by a score of ≥−1 standard deviation when applying the two stricter deficit criteria (1.25 and 1.5 deficit criteria).

For each subsample, proportions (for isolated disorders and the different comorbid disorders) were calculated together with confidence intervals (CI). CIs were calculated using the Wilson/score interval [32], which provides a more reliable coverage than the standard Wald interval and is recommended for small as well as large sample sizes and therefore most suitable for the current analyses (for a critical discussion see [33]).

## Results

### Prevalence rates

**Figure 1:**
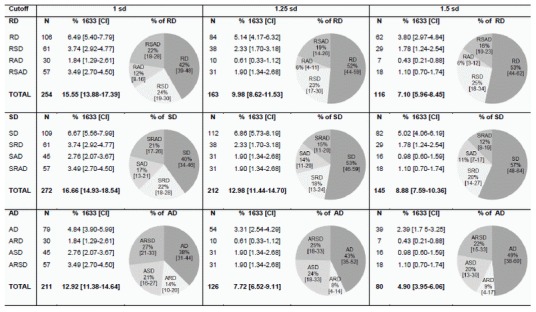
Prevalence rates (N = 1633) for specific learning disorders: isolated and combined deficits in reading, spelling, and arithmetic skills. *Note:* sd = standard deviation; CI = confidence interval; RD = Reading Deficit; SD = Spelling Deficit; AD = Arithmetic Deficit; RSD/SRD = Reading and spelling Deficit; RAD/ARD = Reading and arithmetic Deficit; SAD/ASD = Spelling and Arithmetic Deficit; RSAD/SRAD/ARSD = Reading, spelling and arithmetic Deficit.

**Figure 1** shows the number of children (N) affected in reading (RD), spelling (SD), and arithmetic (AD) and the percentages of isolated and comorbid learning disorders together with confidence intervals for the 1 standard deviation deficit criterion (left column), the 1.25 deficit criterion (middle), and the 1.5 deficit criterion (right column), based on the whole sample (% 1633). The pie charts illustrate how many children (in percentage) have deficits in one learning domain only and how many children have combined learning deficits. Isolated and combined deficits were calculated separately for each learning domain (RD, SD, and AD) based on all children with a specific learning disorder.

**Figure 2:**
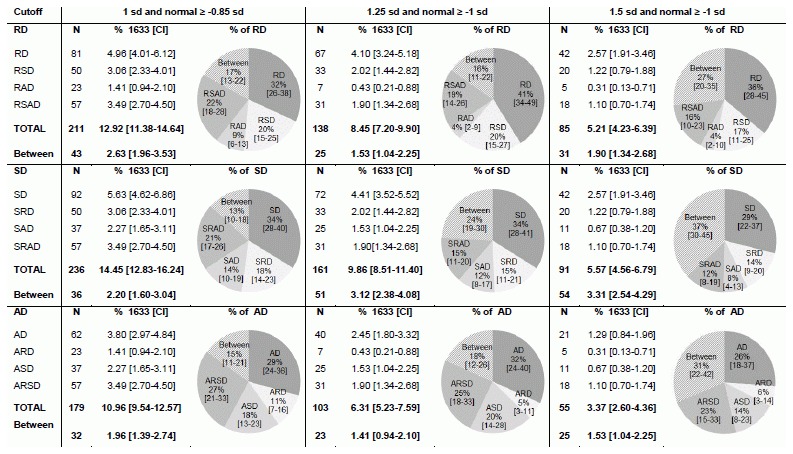
Prevalence rates (N = 1633) for specific learning disorders including cutoff for normal performance. *Note:* Between = children who fulfill deficit criteria for the relevant domain (RD, SD or AD), but score between the deficit criterion and the criterion for normal performance on at least one other measure.

The total number of children identified with RD, SD or AD simply reflects the cutoff criterion used to classify learning problems. The first relevant finding is that, when comparing isolated versus combined learning problems, approximately half of the children showed deficits in a single learning domain, while the other half showed comorbid learning problems. However, the proportion between isolated and comorbid disorders also depends on the cutoff criterion. Comorbid disorders were significantly more frequent than isolated disorders when using the lenient cutoff criterion of 1 standard deviation (RD: 58% comorbid vs. 42% isolated; SD: 60% vs. 40%; AD: 62% vs. 38%; all z-values>3.5, *p*s<.001). In contrast, isolated learning problems were as frequent or more frequent when applying a stricter criterion of 1.5 standard deviations (RD: 47% comorbid vs. 53% isolated, z = 0.9, *p*>.05; SD: 43% vs. 57%, z = 2.4, *p*<.05; AD: 51% vs. 49%, z = 0.3, *p*>.05). Obviously, the absolute number of children with isolated and combined learning disorders decreased when including a criterion for normal performance (**Figure 2**); this is due to a significant number of children (RD: 13%, 16% and 27% for the 1 sd, 1.25 sd and 1.5 sd, respectively; SD: 13%, 24%, and 37%; AD: 15%, 18%, and 31%) who fell in-between the criteria for impaired and normal performance in at least one learning domain and could therefore not be allocated to any of the deficit groups. However, the relation between isolated and comorbid learning disorders was similar to the analysis without a criterion for normal performance. Again, comorbid deficits were significantly more frequent than isolated deficits for the lenient cutoff criterion of 1 standard deviation (RD: 51% vs. 32%; SD: 53% vs. 34%; AD: 56% vs. 29%; z-values = 4.3–5.6, *p*s<.001), while for the stricter criterion of 1.5 standard deviations the proportion between comorbid and isolated deficits was more balanced (RD: 37% vs 36%, z = 0.2, *p*>.05; SD: 34% vs 29%, z = 0.9, *p*>.05; AD: 43% vs. 26%, z = 2.3, *p*<.05).

Within the comorbid disorders, the highest prevalence rates were observed for combined reading and spelling disorder and for deficits in all three learning domains, followed by combined spelling and arithmetic deficits. Importantly, there was a difference between the two literacy measures (reading and spelling) with respect to their association with arithmetic deficits. Deficits in arithmetic and spelling co-occurred more often than deficits in arithmetic and reading (1 sd: 21% vs. 14%, z = 1.9, *p* = .06; 1.25 sd: 24% vs. 8%, z = 3.5, *p*<.001 and 1.5 sd: 20% vs. 9%, z = 2.0, *p*<.05). Results were comparable when including a cutoff criterion for normal performance (1 sd: 18% vs. 11%, z = 2.0, *p*<.05; 1.25 sd: 20% vs. 5%, z = 3.6, *p*<.001 and 1.5 sd: 14% vs. 6%, no statistics due to small subsample).

Next we calculated ratios for the different comorbidity rates by comparing the comorbidity rates that would be expected by chance, based on the base-rates for each disorder, with the comorbidity rates observed in our sample [34]. As evident from **Table 2**, all comorbidity rates were three to five times higher than the rates expected by chance and these ratios increased with stricter deficit criteria from 2.6–2.9 for 1 standard deviation to 4.2–4.9 for 1.5 standard deviations.

**Table 2:** Expected and observed number of comorbid cases for the three cutoff criteria.

Deficit	Cutoff [sd]	Comorbidity rate expected %	Cases expected [E]	Cases observed [O]	Ratio O/E
RD+SD	1	2.59	42	118	2.8
	1.25	1.30	21	69	3.3
	1.5	0.63	10	47	4.7
RD+AD	1	2.01	33	87	2.6
	1.25	0.77	13	41	3.2
	1.5	0.35	6	25	4.2
SD+AD	1	2.15	35	102	2.9
	1.25	1.00	16	62	3.9
	1.5	0.44	7	34	4.9

### Gender ratios

Gender ratios for isolated and comorbid learning disorders are reported in **Table 3**. The observed proportion of boys and girls was compared to the proportion in the representative sample (50.6% male). More boys than girls showed isolated spelling deficits and combined reading and spelling deficits, while more girls were impaired in arithmetic (AD only and AD+SD). No gender differences were observed for RD (RD only and RD+AD) and for the combination of all three learning disorders. Once again reading and spelling deficits differed with respect to their association with AD. While more girls were affected in RD+AD, the gender ratio for SD+AD was balanced.

**Table 3:** Gender ratios for isolated and comorbid learning disorders.

	1 sd		1.25 sd		1.5 sd	
Deficit	% male	*χ^2^*	% male	*χ^2^*	% male	*χ^2^*
RD total	54.7	1.7	54.0	0.7	51.7	0.1
SD total	55.5	2.6	55.2	1.8	55.2	1.2
AD total	40.8	7.9**	38.1	7.4**	37.5	5.0*
RD only	51.9	0.1	56.0	1.0	53.2	0.2
SD only	62.4	6.0*	61.6	5.4**	61.0	3.5
AD only	32.9	9.2**	35.2	4.6**	38.5	1.9
RD+SD	66.7	5.4*	63.2	2.4	62.1	1.5
RD+AD	56.7	0.4	50.0	0.0	42.9	0.2
SD+AD	35.6	4.1*	38.7	1.8	37.5	1.1
RD+SD+AD	47.4	0.2	38.7	1.8	33.3	2.2

*p<.05;

**p<.01 (two-sided).

## Discussion

The current study examined prevalence rates and gender ratios for learning disorders, classified based on DSM-5 criteria. This is one of the few existing studies including all three learning domains (reading, spelling, and arithmetic) and assessing prevalence rates and gender ratios for isolated disorders as well as comorbid learning disorders. Prevalence rates were systematically analysed for different cutoff criteria, including, for the first time, a criterion for normal performance, which allows testing how prevalence rates change when children who just missed the criterion for a second deficit are excluded. Our results showed that including a criterion for normal performance does not affect the overall relation between isolated and comorbid learning disorders. These findings are reassuring because they indicate that previous analyses did not result in a considerable overestimation of isolated deficits.

A main finding of the current study was that comorbid learning disorders occurred as frequently as isolated learning disorders in all three learning domains. Importantly, comorbidity rates were even high when applying the stricter deficit criteria of 1.25 and 1.5 standard deviations (comorbid RD: 48% and 47%; comorbid SD: 47% and 43%; comorbid AD: 57% and 51%) While it could be argued that using a lenient criterion might result in an unreasonable high number of comorbid cases, this overestimation is less likely to occur when using stricter deficit criteria. Indeed, significantly more comorbid cases were identified when using the lenient criterion, while for the two stricter criteria the proportion of isolated and comorbid cases was balanced. The few existing prevalence studies including all three learning domains (**Table 1**) report prevalence rates for isolated and comorbid AD (% of isolated AD among all children with AD calculated based on absolute numbers given in the original paper: [19] 54%; [4] 52%, [23] 30%) which are largely comparable to the rates identified in the current study (38%, 43%, and 49%). However, these studies did not differentiate between reading and spelling deficits when analysing comorbidities with arithmetic problems. This is due to the fact that following ICD-10 classification [35], deficits in arithmetic co-occurring with any literacy deficit (reading and/or spelling problems) are summarized in one and the same diagnostic category (F81.3: mixed disorders of scholastic skills). As a consequence, information about comorbidities between AD+RD versus AD+SD is missing in these studies. The only exception is the prevalence study carried out by Landerl and Moll [4], which differentiated between reading and spelling deficits when analysing comorbidities with AD. In line with the current study, AD co-occurred more often with SD than with RD (Landerl & Moll: 22% vs. 10%; current study: 20% vs. 9%, applying the same deficit criterion of 1.5 standard deviations). Our findings further show that comorbidity rates between AD and SD were comparable for lenient and stricter cutoff criteria (21% for 1 standard deviation vs. 24% and 20% for 1.25 and 1.5 standard deviations). In contrast, the comorbidity rate between AD and RD was highest when using a lenient criterion (14% for 1 standard deviation) and rates decreased for the more stringent criteria (8% and 9%). Stricter criteria are supposed to increase the probability of homogenous samples, including mainly children whose learning problems are neurobiological in origin [36]. Thus, high comorbidity rates in more severe cases, as found between AD and SD, support evidence for a neurobiological basis of the comorbidity. These findings suggest that the processes underlying the relationship between AD and SD might differ from those underlying AD and RD. In a similar vein, Dirks and colleagues [2] found different associations between AD and RD compared to AD and reading comprehension, providing further evidence for dissociations between literacy components (e.g., [14]).

Another distinction that is not made based on ICD-10 classification is to differentiate between isolated RD and combined reading and spelling problems (F81.0). As a consequence information about the proportions of children with isolated RD among all children with RD and isolated SD among all children with SD is missing. In the current study isolated RD was observed in 42–53% (depending on the cutoff criterion) of all children with RD and isolated SD in 40–57% of all children with SD. Landerl and Moll [4] did not directly report proportions of isolated RD and isolated SD; however calculations based on the absolute numbers given in their paper revealed comparable rates of 42% for isolated RD and 46% for isolated SD. Given that reading and spelling skills tap into the same domain and are supposed to be closely related during literacy development [37]–[39], it is surprising that approximately half of the children with reading deficits are not affected in their spelling skills and vice versa. Importantly, dissociations between reading and spelling deficits were still evident even when a cutoff criterion for normal performance was included (**Table 3**), excluding the possibility that children with poor reading skills performed just above the cutoff criterion for poor spelling (and vice versa); instead findings indicate that a large number of children shows a remarkable discrepancy between the two literacy skills (see also [12]). One explanation for the dissociation between reading and spelling deficits is that the cognitive processes underlying reading fluency and spelling might be less similar than those underlying reading accuracy and spelling [40]. At the beginning of literacy instruction, reading (word decoding) and spelling draw on similar processes (e.g., [37], [38]). In order to learn to decode and to spell words accurately, children have to learn the alphabetic principle; they need to be aware that spoken words consist of sounds which are linked to letters or letter groups. The ability to decode words accurately is obviously a precondition of becoming a fluent reader [41]–[43]; however it has been suggested that fluent text reading requires additional skills, such as efficient lexical access and the ability to suppress task-irrelevant lexical information, in order to choose the appropriate target letter or word from the representations activated [44], [45]. Therefore, it can be argued that the cognitive processes underlying fluent reading differ to some extent from those underlying spelling, which could explain why reading fluency and spelling skills can dissociate in a large number of children.

Finally, our findings suggest that gender ratios differ for isolated learning disorders but are balanced for comorbid disorders affecting all three learning domains. We found more girls with problems in arithmetic (overall and with isolated arithmetic disorder) and more boys with problems in spelling. The disproportionate number of boys with literacy problems is in line with the large body of research suggesting that dyslexia is more apparent in boys than in girls [24]. Our results specify previous findings by showing that more boys than girls can be identified with combined literacy deficits (reading and spelling problems) and with isolated spelling disorder. In contrast, gender ratios were balanced for isolated reading problems, a finding that was first reported by Landerl and Moll [4] and recently replicated by Fischbach et al. [19]. The differences in gender ratios observed between RD and SD further support the interpretation that the causes underlying deficits in fluent reading and those underlying spelling are at least to some extent different. Future research will have to replicate these findings and will have to specify whether differences between reading and spelling deficits as well as differences in their association with arithmetic problems are reflected at the neurobiological and genetic level.

As mentioned in the method section, a limitation of the current study is that clinical criteria for classifying learning disorders could not be applied. Thus, future studies will have to clarify whether prevalence rates for isolated and comorbid disorders differ between studies using clinical criteria compared to those based on low-performing samples.

### Practical implications and implications for future research

Our findings have a number of practical implications for assessment and intervention of learning disorders: Given that about half of the children with a specific learning deficit have problems in other learning domains as well, the different learning domains need to be considered during assessment. Performance in reading, writing, and mathematics, should be assessed based on domain specific tests, in order to avoid that children score poorly on a test due to difficulties in other learning domains (e.g., poor performance in maths tests using word problems due to poor reading skills).

With respect to classification systems, DSM-5 takes into account that learning disorders frequently co-occur by using the generic diagnostic term “Specific Learning Disorder”. However, in order to differentiate between isolated and comorbid disorders, practitioners need to specify the diagnosis by providing additional information about the learning domains that are affected (i.e., using specifiers as implemented in DSM-5). Although ICD-10 differentiates between isolated and combined learning disorders, no differentiation is made between the literacy components (reading vs. spelling) and their co-occurrence with mathematics deficits. Furthermore, dissociations between disorders were not only observed between deficits in arithmetic and in literacy skills, but also between deficits in reading and in spelling skills. As a consequence both literacy components need to be assessed to avoid that a large number of children with problems is not identified and therefore does not receive adequate treatment.

With respect to intervention, it seems similarly important to distinguish between isolated and comorbid learning disorders: First, children with comorbid disorder require additional support targeting the comorbid deficit in addition to the initially diagnosed deficit (e.g., numeracy intervention plus reading intervention). Furthermore, these children are impaired in a broad range of skills and their deficits are reported to be more pronounced than in children with deficits in one domain only (e.g., [46]). As a result, children with comorbid disorders will have fewer possibilities to compensate for their deficits, so that strategies applied during intervention need to be attuned to the child’s cognitive profile.

Future research will have to specify associations and dissociations between other literacy components, especially between word decoding and reading comprehension, but also between different aspects of maths, such as maths reasoning and calculation skills. In addition, the current results which focused on the behavioural level raise the question of how associations and dissociations between learning disorders are reflected at the cognitive and neurobiological level. While the core cognitive deficits associated with reading, spelling and arithmetic disorders appear to be specific, one proposal regarding comorbidity between learning disorders is that domain-general cognitive risk factors, such as memory or processing speed deficits [47]–[49] are shared between learning disorders. This could explain why these disorders frequently co-occur. Based on the idea that developmental disorders are best described as the outcome of multiple interacting risk factors (e.g., [17], [18]), future studies will have to specify the risk factors that are specific to a given disorders and those that are shared between learning disorders. Related to this issue is the question of whether the cognitive profiles observed in comorbid cases reflect the sum of the single deficit profiles or whether comorbid cases are characterised by a distinct cognitive profile. The latter may be associated with different risk factors compared to those observed in groups with deficits in one learning domain only.

Cognitive profiles may not only differ between isolated versus comorbid disorders, but may also depend on the chosen cutoff criteria. In line with this idea, Murphy, Mazzocco, Hanich, and Early [50] showed that the cognitive profiles of children with mathematics learning disability scoring below the 10^th^ percentile differed qualitatively from those scoring between the 11^th^ and 25^th^ percentile. For reading disorder it has been shown that children with poor oral language skills in addition to a phonological deficit are more likely to develop severe reading problems compared to children with a phonological deficit only (e.g., [51]). These findings suggest that the behavioural outcome depends on the severity of the underlying core cognitive deficit as well as on co-occurring difficulties. Future research will have to further specify whether applying different cutoff criteria will result in groups that differ not only in terms of degree of the underlying cognitive deficit, but also qualitatively in terms of different cognitive profiles.
